# Longitudinal Changes in Cortical Thickness in Adolescents with Autism Spectrum Disorder and Their Association with Restricted and Repetitive Behaviors

**DOI:** 10.3390/genes12122024

**Published:** 2021-12-20

**Authors:** Valentina Bieneck, Anke Bletsch, Caroline Mann, Tim Schäfer, Hanna Seelemeyer, Njål Herøy, Jennifer Zimmermann, Charlotte Marie Pretzsch, Elke Hattingen, Christine Ecker

**Affiliations:** 1 Department of Child and Adolescent Psychiatry, Psychosomatics and Psychotherapy, University Hospital, Goethe University, Deutschordenstrasse 50, 60528 Frankfurt, Germany; anke.bletsch@gmx.net (A.B.); caroline.mann@kgu.de (C.M.); tim.schaefer@kgu.de (T.S.); hanna.seelemeyer@kgu.de (H.S.); NjalSebastian.Heroey@kgu.de (N.H.); jenny_zimmermann89@web.de (J.Z.); christine.ecker@kgu.de (C.E.); 2 Brain Imaging Center, Schleusenweg 2-16, Haus 95H, Goethe University, 60528 Frankfurt, Germany; 3 Department of Forensic and Neurodevelopmental Sciences, Institute of Psychiatry, Psychology and Neuroscience, King’s College, London SE5 8AF, UK; charlotte.pretzsch@kcl.ac.uk; 4 Institute for Neuroradiology, University Hospital, Goethe University, 60528 Frankfurt, Germany; elke.hattingen@kgu.de

**Keywords:** autism spectrum disorder, cortical thickness, restricted and repetitive behaviors, genetics

## Abstract

The neuroanatomy of autism spectrum disorder (ASD) shows highly heterogeneous developmental trajectories across individuals. Mapping atypical brain development onto clinical phenotypes, and establishing their molecular underpinnings, is therefore crucial for patient stratification and subtyping. In this longitudinal study we examined intra- and inter-individual differences in the developmental trajectory of cortical thickness (CT) in childhood and adolescence, and their genomic underpinnings, in 33 individuals with ASD and 37 typically developing controls (aged 11–18 years). Moreover, we aimed to link regional atypical CT development to intra-individual variations in restricted and repetitive behavior (RRB) over a two-year time period. Individuals with ASD showed significantly reduced cortical thinning in several of the brain regions functionally related to wider autism symptoms and traits (e.g., fronto-temporal and cingulate cortices). The spatial patterns of the neuroanatomical differences in CT were enriched for genes known to be associated with ASD at a genetic and transcriptomic level. Further, intra-individual differences in CT correlated with within-subject variability in the severity of RRBs. Our findings represent an important step towards characterizing the neuroanatomical underpinnings of ASD across development based upon measures of CT. Moreover, our findings provide important novel insights into the link between microscopic and macroscopic pathology in ASD, as well as their relationship with different clinical ASD phenotypes.

## 1. Introduction

Autism Spectrum Disorder (ASD) is a neurodevelopmental condition characterized by impairments in (1) social communication and interaction, (2) repetitive and restricted behaviors and interests, and atypical sensory responses (DSM-5, 2013; [1]). However, the clinical phenotype of ASD is highly heterogeneous both within (e.g., across age) and between (e.g., in terms of the severity and profile of core and associated symptoms [2]) individuals. Similarly, there exists large inter-individual heterogeneity in the neurobiology of ASD, including in the neuroanatomy and genetics [2,3]. For example, while neuroimaging studies in ASD agree on an atypical developmental trajectory of brain maturation in affected individuals ([2,4,5]), the reported spatial distribution of these differences reveal a high level of diversity across development [6]. Moreover, ASD has been linked to hundreds of genetic variants. This suggests that ASD is not a single gene disorder, but has a highly complex (poly)genetic architecture [7]. Combined, these findings highlight inherent difficulties in linking differences in the brain structure to etiological factors, and determining their impact on clinical outcomes in ASD.

While a wealth of studies have explored the neural networks underpinning ASD [8], the reported differences vary in terms of the specific patterns and sign (i.e., direction) [2]. Moreover, the patterns of differences vary for different morphometric features, including volumetric and geometric measures of the neuroanatomy [9,10,11]. One of the morphological parameters in which atypicalities in ASD have been reported most consistently, is in the cortical thickness (CT); i.e., the closest distance between the outer (i.e., pial) and inner (i.e., white matter) boundary at each vertex on the tessellated surface [12]. It is well established that the developmental trajectory of CT in ASD deviates from the neurotypical trajectory, even though reports vary with regard to the direction of the difference. For example, in a longitudinal study by Zielinski et al. (2014), ASD was characterized by an overgrowth of the cortical mantle during early childhood, followed by an accelerated decline in mid-childhood, and a phase of ‘normalization’ during adulthood [5]. Contrarily, Wallace et al. (2010) reported an accelerated—rather than decelerated—cortical thinning during adulthood in a cross-sectional sample of ASD individuals [11]. These studies highlight some of the inconsistencies of neuroanatomical findings derived from longitudinal and cross-sectional studies designed to investigate age-related changes in the neuroanatomy of ASD. Hence, further longitudinal studies are required to replicate and validate these earlier findings.

Despite these differing results, studies agree that fronto-temporal and fronto-parietal brain regions [13,14,15] are particularly affected in ASD, and display the most atypical neurodevelopmental trajectory relative to other brain regions. Overall, neuroanatomical differences in ASD have been reported in (1) fronto-temporal and fronto-parietal brain networks including the medial, orbitofrontal (OFC) and inferior frontal (IFG) cortices, the posterior parietal cortex, the superior temporal sulcus (STS) and the fusiform gyrus; (2) limbic brain regions such as the amygdala–hippocampal complex, the thalamus, and cingulate regions; (3) the fronto-striatal circuitry including parts of the basal ganglia, the anterior cingulate cortex (ACC) and the dorsolateral prefrontal cortex (DLPFC); and (4) the cerebellum [2,8]. Most of these brain regions are integral parts of the so-called ‘social brain’ that underpins higher socio-cognitive functioning [16]. However, less is known about the neuroanatomical underpinnings of restricted and repetitive behaviors (RRBs), which, with the most recent edition of the DSM-V, have only recently become the focus of attention. [1]. Emerging research suggests that the severity of repetitive behaviors in ASD may be correlated with differences in subcortical structures, particularly in parts of the fronto-striatal circuitry (e.g., the volume of the caudate nucleus in the basal ganglia [17,18]). However, few studies have investigated the association between repetitive behaviors and cortical neuroanatomy (e.g., CT) across time. Due to the crucial role of the cortex in fronto-striatal pathways [19], it is important to not only assess brain–behavior correlations at a subcortical level, but to furthermore analyze changes in cortical measures and their impact on fronto-striatal connectivity.

Therefore, in this study we compared changes in the CT of children and adolescents with ASD to those of typically developing (TD) controls, and examined the relationship of CT differences with variations in repetitive behavior (as measured by the Restricted and Repetitive Behavior Scale-Revised (RBS-R) [20,21]) over time. We focused on RRB because most neuroanatomical studies to date have examined brain–behavioral correlations with social impairments, leaving RRB largely unexplored. Furthermore, RRBs are known to be associated with sensory symptoms (e.g., hyperresponsive behaviors; [22]), which have been highlighted as promising new candidates for subtyping ASD individuals [23]. In contrast to most previous studies that have examined neurodevelopmental trajectories in CT during early to mid-childhood [4,13,24,25,26,27], we focused on the developmental changes in CT during late childhood and adolescence based on a narrower age range (11–18 years). This allowed us to investigate a crucial stage in development during which CT growth curves in ASD and TD controls typically overlap [5], even though ASD symptoms persist across the human life span. Last, using the spatial gene expression data provided by the Allen Human Brain Atlas (AHBA; [28]), we tested the hypothesis that brain regions with atypical CT development during adolescence in ASD are enriched for genes that have previously been linked to ASD on the genetic and transcriptomic level. 

## 2. Materials and Methods

### 2.1. Participants

**Figure 1:**
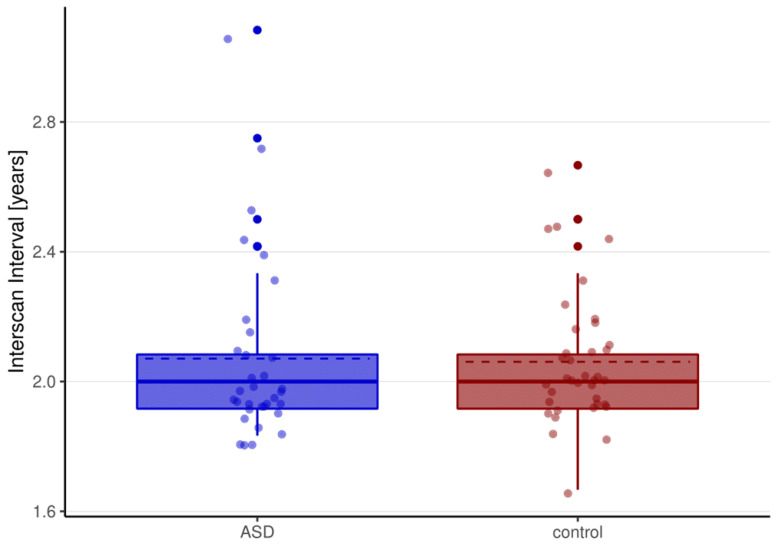
Interscan Interval (ISI) of the ASD and control group. Time between the two scanning timepoints in the ASD group (blue) and the control group (red). Dashed lines represent mean scores of ISI, solid lines represent the median scores of ISI.

This study used data provided by an ongoing longitudinal study examining brain development during adolescence in individuals with ASD and in TD controls at two timepoints separated by ~2 years (see Figure 1). The total sample consisted of *n* = 70 individuals of between 11 and 18 years at timepoint 1, out of which *n* = 33 participants had a diagnosis of ASD, and *n* = 37 were TD controls. Groups were matched for age, sex, and full-scale IQ (FSIQ; see Table 1). ASD was assessed using gold-standard diagnostic tools, i.e., the German version of the Autism Diagnostic Interview-Revised (ADI-R; [29,30]) and the second edition of the Autism Diagnostic Observation Schedule (ADOS-2; [31,32]). Repetitive behaviors were examined in all participants using the German version of the Repetitive Behavior Scale-Revised (RBS-R) at both assessment timepoints [20,21]. In accordance with Kästel et al. (2014), we used a four-factor model, containing persistent (F1), stereotyped (F2), self-injurious (F3), and compulsive (F4) behaviors to compute the RBS-R subscales. A full list of inclusion and exclusion criteria and the clinical characteristics of the sample is provided in the Supplementary Materials (see Supplementary Methods 1 and Supplementary Table S1). All participants (guardians of participants below 18 years of age), gave informed written consent. The study was approved by the Ethics Committee of the Faculty of Medicine of Goethe University, Frankfurt.

### 2.2. MRI Data Acquisition

All MRI data were acquired at the Brain Imaging Centre (BIC), Frankfurt using a contemporary MRI scanner operating at 3 Tesla (Magnetom Trio, Siemens Medical Systems, Erlangen, Germany). High-resolution structural ADNI MPRAGE sequences were acquired with full head coverage using an 8-channel head coil (slice thickness = 1.0 mm, in-plane resolution = 1.0 × 1.0 mm^2^, repetition time (TR) = 2300 ms, echo time (TE) = 2.2 ms, flip angle = 9°, field of view = 26.5 cm, slice number = 176).

### 2.3. Cortical Surface Reconstruction Using FreeSurfer

Image processing and cortical reconstruction for all MRI scans was performed using FreeSurfer v6.0.0 software (https://surfer.nmr.mgh.harvard.edu/, accessed on 11 November 2020). Here, models of the cortical surface are created for each T1-weighted image, i.e., one image per subject and timepoint, by implementing well validated and fully automated procedures. These have been extensively described in previous studies [34,35,36]. The reconstructed scans were further processed using FreeSurfer’s longitudinal stream to derive estimates of the vertex-wise change in CT [34]. Here, an unbiased within-subject template (i.e., ‘base’ image) is initially created using cubic spline interpolation reflecting the average anatomy of each subject across time to reduce the confounding effect of inter-individual morphological variability [37,38]. Subsequent processing steps of the single timepoints, including skull stripping, Talairach transforms, and atlas registration, are then based upon this common information from the within-subject template. This significantly improves the reliability and statistical power [34]. More information on the quality assurance procedures is provided in the Supplementary Materials (see Supplementary Methods 2). CT was calculated based on the longitudinal scans as the closest distance from the outer (i.e., pial) to the inner (i.e., white matter) boundary at each vertex on the tessellated surface [12]. Vertex-wise estimates of the longitudinal CT change were expressed as the Symmetrized Percent Change (CT_spc_) within the framework of FreeSurfer’s longitudinal stream (https://surfer.nmr.mgh.harvard.edu/fswiki/LongitudinalTwoStageModel, accessed on 11 November 2020), which is calculated as

$$CT_{\mathrm{spc}}=100*\frac{\mathrm{rate}}{\mathrm{average}}$$

With the $\mathrm{rage}=0.5*\left(CT_{T1}+CT_{T2}\right)$, and $\mathrm{rate}=CT_{T2}-(\frac{CT_{T1}}{ISI}$). Hence, this parameter represents change the in CT of individuals from T1 to T2 at each vertex, while accounting for the average thickness across the cortex and intra-individual noise. Vertex-wise estimates of CT_spc_ were registered to a common space surface template (i.e., fsaverage in FreeSurfer) and smoothed using a 10-mm surface-based smoothing kernel, to increase the ability to detect population changes.

### 2.4. Statistical Analyses 

Surface-based statistical analyses were performed using the SurfStat toolbox (http://www.math.mcgill.ca/keith/surfstat, accessed on 11 November 2020) for Matlab (R2021a; MathWorks), and R (version 4.0.5) for the Statistical Computing (www.r-project.org, accessed on 16 December 2020). Missing data in RBS-R (maximum 3 out of 43 items per person) was input by predictive mean matching using the ‘mice’ package in R [39]. Between-group differences in age at both timepoints, ISI, sex, FSIQ, handedness, ASD symptom severity, total brain measures at both timepoints and their change, were assessed via *t*-test or *χ*^2^-test (see Table 1). We initially applied a step-up model selection procedure using a nested model comparison to identify the general linear model (GLM) that best fitted our CT data (for more information see Supplementary Methods 3). Based on the best-fitting model, vertex-wise between-group differences in CT_spc_ (*Y*) were subsequently examined by applying a GLM with the diagnostic group and sex as the fixed-effects factors, and the linear and quadratic age at T1, FSIQ, and ISI as the continuous covariates, i.e.,

(1) $$\gamma_{i\;}=\beta_{0}+\beta_{1}\mathrm{Group}+\beta_{2}\mathrm{Sex}+\beta_{3}{\mathrm{Age}}_{T1}+\beta_{4}{\mathrm{Age}}_{T1}^{2}+\beta_{5}\mathrm{FSIQ}+\beta_{6}\mathrm{ISI}+\varepsilon_{i}$$

where *ɛ_i_* is the residual error at vertex *i*. Between-group differences were estimated from the coefficient *β*_1_, and normalized by the corresponding standard error.

In a second analysis step, we examined the association between the developmental change in CT (CT_spc_) and developmental changes in the severity of general autism symptoms and repetitive behaviors across adolescence, which was quantified as the intra-individual change in the RBS-R total score from T1 to T2 (ΔRBS-R_(T2-T1)_). The employed GLM was derived using a step-up model selection procedure that assessed the goodness-of-fit upon inclusion of the ΔRBS-R_(T2-T1)_ and the ΔRBS-R_(T2-T1)_-by-group interaction (see Supplementary Methods 3). Based on the model comparison, the following model was fitted

(2) $$\gamma_{i}=\beta_{0}+\beta_{1}\mathrm{Group}+\beta_{2}\mathrm{Sex}+\beta_{3}{\mathrm{Age}}_{T1}+\beta_{4}{\mathrm{Age}}_{T1}^{2}+\beta_{5}\mathrm{FSIQ}+\beta_{6}\mathrm{ISA}+\beta_{7}\Delta {\mathrm{RBSR}}_{\left(T2-T1\right)}+\varepsilon_{i}$$

We did not covary for the mean CT across the cortex, as this is already accounted for in the computation of CT_spc_. Since variance in ΔRBS-R_(T2-T1)_ was mainly driven by individuals within the ASD group (see Supplementary Figure S1), brain-behavioral correlations were examined both in the total sample and within the ASD individuals. The main effect of ΔRBS-R_(T2-T1)_ on CT_spc_ was then calculated from the respective coefficient *β*_7_. In the ASD group, associations between regional deviations from the neurotypical developmental trajectory of CT and developmental changes in autism symptoms and repetitive behaviors were further examined via Pearson correlation. 

In all GLMs, the continuous covariates were mean centered across groups to improve interpretability of the coefficients. In the subanalysis of individuals with ASD, mean centering was performed across all included participants with ASD. Corrections for multiple comparisons across the whole brain were performed using ‘random field theory’ (RFT)-based cluster analysis for nonisotropic images with a cluster-forming and cluster-based significance threshold of *p* < 0.05 (2-tailed; [40]).

### 2.5. Gene Expression Decoding Analysis 

**Figure 2:**
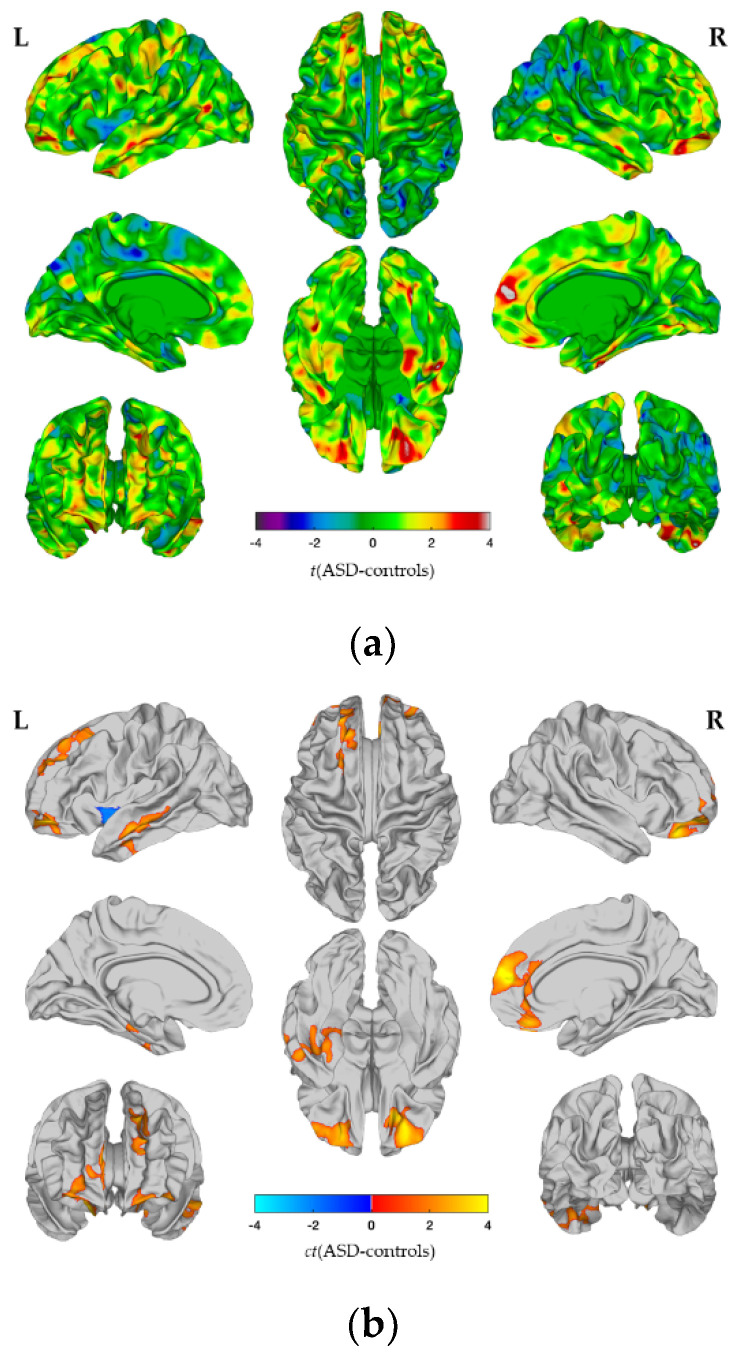
Vertex-wise between-group differences in cortical thickness (CT). Vertex-wise between-group differences in Scheme 1 to T2, i.e., the rate of CT change with respect to the average CT, in individuals with autism spectrum disorder (ASD) compared to neurotypical controls. (**a**) *t*-test statistic for the contrast ASD minus controls (unthresholded); (**b**) Positive clusters (orange to yellow) indicate significantly less cortical thinning, negative clusters (blue to cyan) indicate significantly stronger cortical thinning in ASD (RFT-based cluster corrected, *p* < 0.05, two-tailed). Abbreviations: L: left hemisphere, R: right hemisphere.

To identify the potential genetic underpinnings of the observed neuroanatomical findings, we used the spatial gene expression data provided by the Allen Human Brain Atlas (AHBA; [28]) to perform a gene expression decoding analysis (GEDA; [41]). Here, a total of 20,787 protein coding genes were statistically tested for a spatial pattern of expression that was similar to the spatial pattern of neuroanatomical differences highlighted by the vertex-wise analyses of CT, e.g., the *t*-map for the between-group difference in CT_spc_. The resulting gene list was thresholded at *p* < 0.05 (see Figure 2a; for more information on the methodological approach further see Supplementary Methods 4). This liberal threshold was selected as this analysis did not constitute a hypothesis test per se, but rather a selection step to provide a list of potential candidate genes. Subsequently, the resultant gene list was tested for enrichment using lists of genes that have previously been implicated in ASD by genetic and transcriptomic studies [42,43,44,45,46]. At the genetic level, this included ASD risk genes with de novo and rare variants [44], and GWAS-significant ASD risk genes with common variants [47]. At the transcriptomic level, this included genes that are (i) differentially expressed (i.e., upregulated or downregulated) in postmortem cortical tissue in ASD [43], and in specific neuronal cell types in ASD [45], and (ii) genes of differentially expressed coregulated modules in ASD [42,48]. We also included the ASD-gene list compiled by the SFARI gene database (categories S,1,2,3 downloaded 11 November 2020 from https://gene.sfari.org/).

**Figure 3:**
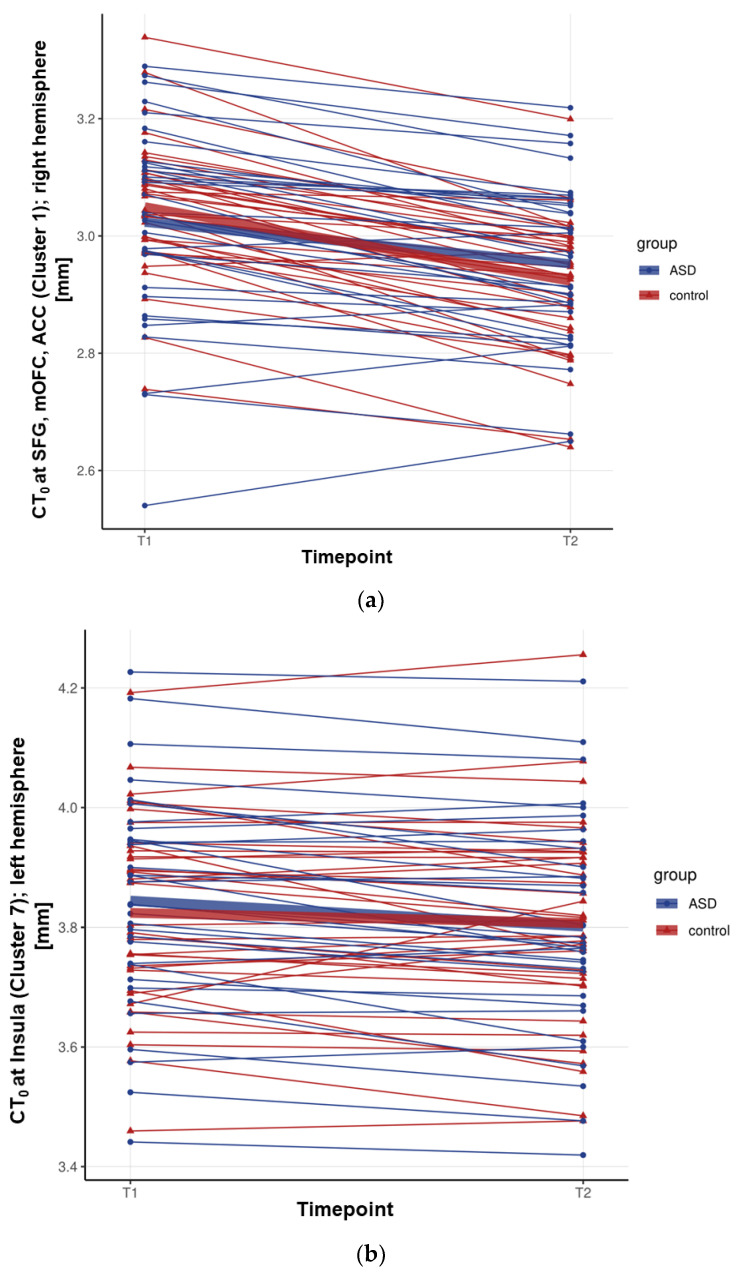
Cluster-wise mean Cortical Thickness (CT) at timepoint 1 (T1) and timepoint 2 (T2). Cluster-wise estimates of the mean CT of individuals at T1 and T2 for the clusters in which a significant between-group difference was observed between individuals with autism spectrum disorder (ASD) (marked in blue) and typically developing (TD) controls (marked in red) in the main analysis, i.e., the main effect of the group for a developmental change in CT. Displayed are two clusters, (**a**) cluster 1 is an example of the first six clusters out of seven, in which, overall, developmental cortical thinning was more pronounced in TD controls as compared to individuals with ASD; and (**b**) cluster 7, which was the only cluster, where individuals with ASD, overall, showed more pronounced developmental cortical thinning relative to TD controls. Abbreviations: SFG: superior frontal gyrus; mOFC: medial orbital frontal cortex; ACC: anterior cingulate cortex.

Furthermore, we examined the *t*-map associated with the main effect of RBS-R variation over time (see Figure 3a) for enrichment of genes previously linked to repetitive behavior in ASD [46]. All enrichment tests were performed using the GeneOverlap package in R (10.18129/B9.bioc.GeneOverlap, accessed on 20 January 2021), which generated enrichment odds ratios (OR), hypergeometric *p* values, and FDR-corrected *p* values (*p_adj_*). Only comparisons with *p_adj_* < 0.05 were interpreted further (more details on the methodological approach of the enrichment analysis are provided in the Supplementary Methods 5). 

## 3. Results

### 3.1. Subject Demographics

There were no significant differences between individuals with ASD and TD controls in their age (t(66) = 1.25, *p* = 0.22), ISI (t(58) = 0.17, *p* = 0.87), FSIQ (t(64) = −1.91, *p* = 0.06), or in the distribution of sex (χ2(1)  < 0.001, *p* = 1.00) and handedness (χ2(1)  = 0.025, *p* = 0.87). Furthermore, there was no significant difference in the mean CT at T1 or T2 (T1 (t(61) = −1.15, *p* = 0.26); T2 (t(68)_meanCTT2_ = −0.81, *p* = 0.42)), or in the overall CT change between T1 and T2 (∆(T2-T1): t(62) = 0.89, *p* = 0.38). However, we observed a significant between-group difference in the total brain volume (T1: t(66)_CVT1_ = −2.6, *p* < 0.05; T2: t(67)_CVT2_ = −2.49, *p* < 0.05; ∆(T2-T1): t(68)_∆CV_ = −0.2, *p* = 0.85) and total surface area (SA) at both timepoints (T1: t(66)_SAT1_= -2.33, *p* < 0.05; T2: t(66)_SAT2_ = −2.28, *p* < 0.05; ∆(T2-T1): t(68)_∆SA_ = −0.16, *p* = 0.88). There was no significant difference in the total ∆CV or total ∆SA over time. For further detailed statistical details, see Table 1.

#### Intra-Individual Differences in RBS-R Total Severity Scores over Time

The majority of ASD individuals (60.1%) showed a decrease in total RBS-R scores between T1 and T2 (maximum decrease [maxD] = −47), 33.3% had higher RBS-R scores at T2 (maximum increase [maxI] = +35), and 6% did not change between T1 and T2 (ΔRBS-R_total severity_(T2-T1) = 0). In contrast, most TD controls (57%) showed no change in RBS-R over time. Here, 21.6% had a decrease (maxD = −8), while an increase in the RBS-R total score was observed in 21.2% (maxI= +8; see Supplementary Table S2). 

### 3.2. Between-Group Differences in CT_spc_

In both groups, we observed widespread cortical thinning across the cortex with increasing age (i.e., between T1 and T2). However, individuals with ASD showed reduced (i.e., decelerated) cortical thinning relative to TD controls, particularly in the fronto-cingulate and temporal regions. More specifically, individuals with ASD showed significant reductions in cortical thinning in the bilateral superior frontal gyrus (approximate Brodmann areas [BA] 4/6/8/10), the rostral middle frontal gyrus (BA 9/46), the right medial orbital frontal cortex (BA 11/32), the rostral anterior cingulate cortex (BA 24/33), the left pars orbitalis (BA 47), the left inferior temporal (BA 20), the middle temporal gyrus (BA 21), and the fusiform and parahippocampal gyrus (BA 17–19/37). An increased cortical thinning in individuals with ASD as compared to TD controls was observed in the left insula exclusively (BA 13) (see Figure 2a,b, Figure 3a,b and Table 2).

### 3.3. Brain–Behavioural Correlations

To examine the association between cortical thinning and variability and measures of symptom severity over time, we correlated the mean CT_spc_ of individuals from clusters with significant between-group differences, with the severity of autism symptoms at T1 (i.e., total and subdomain scores of the ADOS, ADI-R, and RBS-R; see Supplementary Figure S2). We observed a significant negative correlation between CT_spc_ in the right lateral orbital frontal cortex and right rostral middle frontal gyrus (cluster 2 in Table 2) and a change in self-injurious behavior as measured by the RBS-R. We also observed a significant negative correlation between CT_spc_ and a change in stereotypic behavior in the left middle temporal gyrus (cluster 6 in Table 2). Hence, a reduction in cortical thinning was associated with stable or worsening symptom severity from T1 to T2. However, none of these correlations remained significant following FDR correction for multiple comparisons; hence, our findings should be interpreted as preliminary. 

### 3.4. Association between a Longitudinal Change in CT and a Change in Repetitive Behaviors

**Figure 4:**
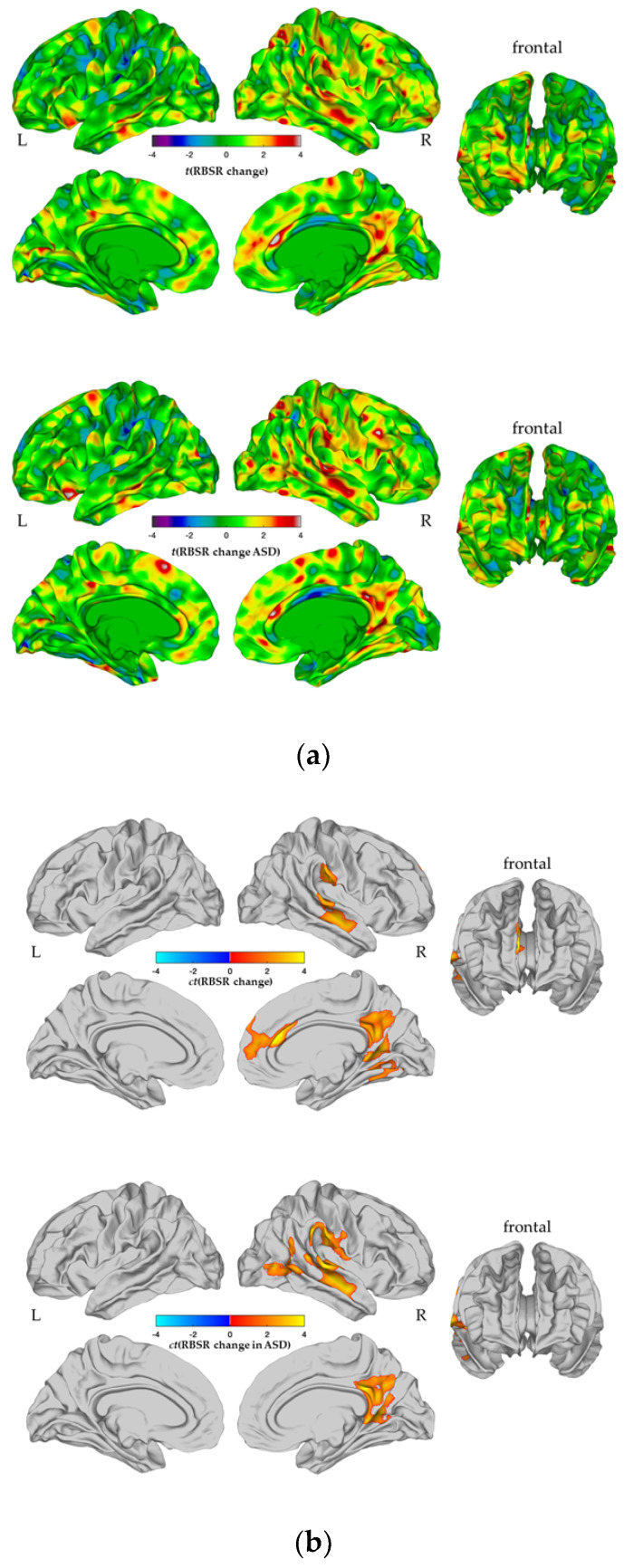
Main effect of a change in restricted and repetitive behaviors (RRBs) on vertex-wise differences in the change in Cortical Thickness (CT_spc_). A significant main effect of developmental changes in the total severity in RRBs was quantified as the difference in the total severity on the RBS-R between timepoint 1 and timepoint 2 ΔRBSR_total severity_(T2-T1)], on vertex-wise differences in the developmental change in CT (CT_spc_) across the total sample [upper figures in (**a**) and (**b**)], and only within individuals with ASD [bottom figures in (**a**) and (**b**)]. The unthresholded t-maps are displayed in (**a**) and the random field theory (RFT)-based, cluster corrected (*p* < 0.05, 2-tailed) difference maps, determined following multiple comparisons are in (**b**). Associations between increases in the RBS-R severity from T1 to T2 with vertex-wise changes in CT were marked in yellow to red (left panel), respectively, and in red to yellow (right panel), and associations between decreases in RBS-R severity from T1 to T2 with vertex-wise changes in CT were marked in cyan to purple (left panel), respectively, and in blue to cyan (right panel). Abbreviations: L: left hemisphere, R: right hemisphere. The green to red color scale indicates regions with an increased CT in the ASD group relative to the control group, while the blue to violet color scale indicates vertices with a decreased CT in the ASD group relative to the control group.

When examining the main effect of RBS-R_Δ_ in the entire sample (i.e., individuals with ASD and TD controls), we identified six clusters where a change in RBS-R was significantly associated with a change in CT (RFT-based cluster correction, *p* < 0.05, 2-tailed). These clusters included the right superior frontal gyrus (BA 4/6/8/10), the caudal anterior cingulate cortex (BA 24/33), the supramarginal gyrus (BA 40), the precuneus cortex (BA 7/31), the isthmus cingulate cortex (BA 29/30), the superior temporal gyrus (BA 22/42), the middle temporal gyrus (BA 21), as well as the lingual and fusiform gyri (BA 17/18/19/37) (see Supplementary Table S3). In these brain regions, cortical thinning between T1 and T2 was associated with less severe RBS-R symptoms over time (Supplementary Figure S4). These associations did not differ significantly by group, i.e., including an RBS-R change-by-group interaction term did not significantly improve the model fit (see Supplementary Figure S3). Because the main effect of RBS-R change on CT_spc_ was mainly driven by variability within ASD individuals, we also repeated the analysis within the ASD group. Here, a reduction in RBS-R was related to a decrease in CT in three significant clusters, including the right superior temporal gyrus (BA 22/42), the middle temporal gyrus (BA 21), the inferior parietal cortex (BA 39), the banks superior temporal sulcus (BA 22/42), the lateral occipital cortex (BA 17–19), the transverse temporal cortex (BA 41), the precuneus cortex (BA 7/31), the isthmus cingulate cortex (BA 29/30), the supramarginal gyrus (BA 40), the postcentral gyrus (BA 1/2/3), and the right insula (BA 13) (see Figure 4a,b). 

### 3.5. Gene Set Enrichment Analyses

**Figure 5:**
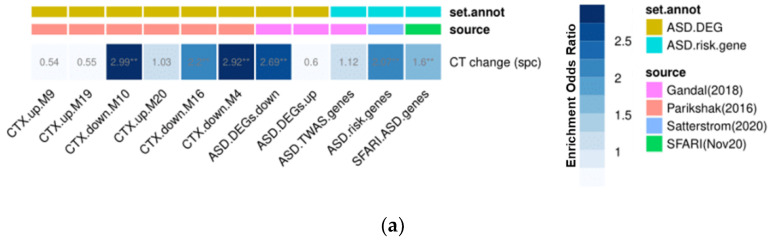

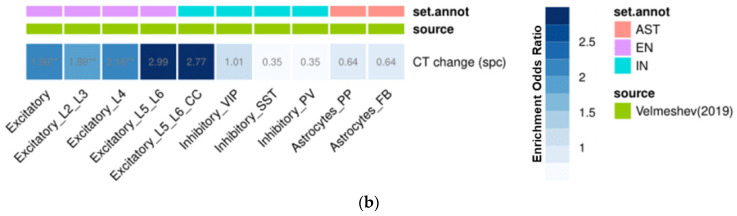
Genomic and cellular underpinnings of neurodevelopmental deviations in the change in Cortical Thickness (CT_spc_) in ASD. The enrichment analysis of genomic underpinnings of the symmetrized percent change in CT (CT_spc_) was based on the *t*-map of statistical between-group differences in the developmental change in CT during adolescence in individuals with autism spectrum disorder (ASD), relative to neurotypical controls (Figure 2a) and various gene sets. (**a**) The significant odds-ratios (OR) at an FDR-rate *p* value of < 0.05 resulting for genes expressed in the *t*-map. Gene sets were subdivided into sets with differential gene expression in ASD (ASD.DEG) and into sets containing ASD risk genes (ASD.risk.gene). Gene set annotation (set.annot) and labeling were determined by their original publication [42,43,44,45]. Abbreviations: up: upregulated expression in ASD, down: downregulated expression in ASD, CTX: cortex, DEG: differential gene expression, **: significant odds-ratios at an FDR-rate *p* value of < 0.05. (**b**) The set with an enrichment of genes particularly expressed in the upper layer cortex neurons and crucial for brain development, as reported by Velmeshev et al. (2019). Annotations were defined by the three cell types: astrocytes (AST), excitatory neurons (EN), and inhibitory neurons (IN). Abbreviations: L2–6: cortical layers, CC: cortico-cortical, SST: somatostatin, PV: parvalbumin, PP: protoplastic, FB: fibrous.

To link the observed differences in the CT trajectory in ASD to the potential genetic underpinnings, we performed a gene expression decoding analysis of our main output maps. The t-map of between-group differences in CT_spc_ (see Figure 1a) was significantly correlated with the pattern of expression of N = 2589 genes (nominal *p* < 0.05). Within this gene set, we found an enrichment for ASD candidate genes, and for genes that are differentially expressed during childhood and adolescence in ASD (see Figure 5a). More specifically, the t-map showed an enrichment for gene coexpression modules that are known to be downregulated in ASD, namely ASD.DEG.down [43], CTX.down.M4 [42], CTX.down.M10 [42], and CTX.down.M16 [42]. According to the international data base of the bioinformatics initiative, ‘Gene Ontology’ (GO), these coexpression modules represent genes involved in receptor signaling (ASD.DEG.down; [43]) and synaptic transmission (CTX.M4, CTX.M10, CTX.M16; [42]) [49,50]. We found no enrichment of the expression modules known to be upregulated in ASD. Furthermore, we observed an enrichment for ASD risk genes known to affect synaptic signaling, both in excitatory and inhibitory neurons [44] (see Figure 5a). We also performed a cell-type enrichment analysis for the t-map of between-group differences in CT_spc_. We observed a significant enrichment for genes that are dysregulated in excitatory neurons in cortical layers L2/L3 and in L4 synaptic function and transcription factors [45] (see Figure 5b). The odds ratios for all modules and adjusted *p* values are displayed in Figure 5. 

We also tested for an enrichment of gene sets by examining the main effect of RBS-R change in ASD (see Figure 3a), which revealed a set of N = 711 significant genes. However, this gene set was not significantly enriched for genes that have previously been associated with RBS by Tao et al. (2016; [46]; see Supplementary Figure S6).

## 4. Discussion

The aim of this study was to compare the longitudinal neurodevelopmental trajectories of CT in adolescents with ASD to those in TD controls, and to examine their association with intra-individual variation in repetitive behaviors. Moreover, to move towards bridging the gap between macroscopic and microscopic pathology, we examined the spatial patterns of between-group differences in CT for enrichment in (i) genes known to be associated with ASD, and (ii) genes that have previously been linked to repetitive and stereotyped behavior. We identified significant between-group differences in CT_spc_, particularly in frontotemporal regions and the cingulate cortex. These brain regions were also enriched for ASD risk genes, and gene expression modules that are known to be downregulated in the cortex of ASD individuals. Additionally, we observed a significant enrichment for genes underpinning cell transmission processes in specific cell types, particularly in the excitatory neurons of the L2, L3 and L4 cortical layers [45]. Taken together, our findings suggest that differences in the development of CT in ASD are associated with genetic variations in the genes known to be implicated in this condition. Moreover, our study revealed an association between developmental changes in CT (in fronto-temporal, fronto-parietal, and cingulate regions) and intra-individual changes in the severity of repetitive behaviors across adolescence in ASD individuals. Hence, our study extends previous reports of atypical brain development in ASD and links these developmental differences to specific autism symptoms.

In contrast to previous studies examining CT development in ASD [13,14,27,51], we observed a reduced cortical thinning in the ASD group compared to the TD controls. This is line with the fact that, depending on the examined age group, in ASD the existing structural neuroimaging studies of regional developmental differences in CT in cross-sectional and longitudinal samples remain highly inconsistent. Further longitudinal studies are hence required to replicate, validate, and add onto these earlier findings. While some studies report an increased (i.e., accelerated) thinning, others report decreased (i.e., slowed down) thinning of the cortex in ASD over time [2]. More specifically, during childhood and adolescence, cross-sectional studies mostly reported enhanced thinning in ASD relative to the neurotypical trajectory [11,15,26], although the importance of subdividing the neurodevelopmental trajectory into different developmental stages has also been noted. For example, one prior longitudinal study classified individuals into distinct age groups [5]. Here, the period of early childhood was marked by cortical thickening, followed by accelerated thinning in adolescence, and decelerated thinning in early adulthood. Hence, the CT growth curves of ASD and TD controls intersected between childhood and adolescence (10–20 years), during which time no significant differences were observed. Based on these findings, CT differences in the age range we examined in our sample (i.e., 11–18 years) could be expected to ‘pseudonormalize’ in ASD, i.e., few or no differences between groups might be observed when examining between-group differences at T1 and/or T2 [5]. Nonetheless, we observed significant differences in CT development between these times points; i.e., we observed one cluster with an accelerated decrease in CT (the left insula), and several clusters with decelerated cortical thinning in ASD (e.g., in the right and left fronto-temporal regions and the right cingulate cortex). Such discrepancies with other studies might be partially explained by differences in the sample characteristics and analytical approaches. For example, in comparison to Zielinski et al. (2014), the age range examined in our study was narrower (11–18 years vs. 3–36 years, respectively), and we also included female participants [5].

Previous neuroimaging studies examining brain development during childhood and adolescence have characterized the developmental trajectory of brain maturation as an ‘inverted U-shape’ that reaches its maximum during childhood/early adolescence (e.g., based on measures of cortical volume and the grey-white matter tissue contrast) [25,52]. The same curve characteristics have been reported for the developmental trajectory of CT, albeit with earlier peaks (males = 8.6 years, females = 8.4 years), followed by cortical thinning [53]. Consequently, cortical thinning during late childhood/early adolescence is commensurate with maturational processes during this stage of development, so the reduced cortical thinning we observed in our sample might suggest a less rapid or stagnant maturation in ASD. On the cellular level, cortical thinning has also been related to experience-dependent (i.e., learning dependent) synaptic pruning. For example, it is known from histological studies, that the synaptic density in the middle frontal gyrus, where we found less cortical thinning in ASD, shows a postnatal increase in density until the age of around 7 years, followed by a period of synaptic pruning until early adulthood [54]. Synapse elimination is important for the development of complex neural systems, and is paralleled by cortical thinning during brain development [55,56]. Our findings indicate that the cortex in ASD might mature more slowly in specific regions of the brain. Decelerated cortical thinning in ASD during adolescence has also been reported in a study by Raznahan et al. (2010), particularly in the left middle temporal and right superior frontal gyrus [57]. In sum, the macroscopic differences we observe in the brain in ASD might point towards specific underlying mechanisms, such as selective synaptic elimination and the arborization of dendrites and axons, which may be dysregulated in ASD.

Atypical brain development in ASD has previously been related to the severity of ASD-related symptoms and behaviors [8]. However, most studies to date have focused on the relationship between atypical cortex development and social impairments in ASD, commonly measured by ADOS and SRS [58,59]. Meanwhile, investigations of the relationship between neuroanatomy and repetitive/restricted behaviors (RRBs) in ASD remain under-represented in the literature. In our study, we addressed this issue by adding a second analysis stream, where we examined the impact of developmental changes in CT on the severity of RRBs, which are also linked to sensory symptoms that are emerging as promising candidates for parsing heterogeneity in autism [23]. On the behavioral level, RRBs can be divided into ‘repetitive sensory motor’ (lower-level) and ‘insistence on sameness’ (higher-level) behaviors. Stereotyped movements and the repetitive use of specific objects define the former, and ritualistic habits and insistence on well-established routines the latter [60]. Lower-level RRBs (including self-injurious behavior) occur more frequently in younger children with ASD, and in those with lower levels of intelligence [61,62]. This was also observed in our sample, where ASD individuals showed minimal self-injurious behavior (see Supplementary Figure S1) [21,63]. Moreover, in agreement with other studies, we found that in the severity of RRB symptoms, there was a decrease in the total severity score between the two examined timepoints in ASD, which is common within this age range [21,63]. Several studies have reported significant correlations between the severity of repetitive behaviors and measures of brain anatomy in ASD. To date, such brain–behavior correlations have mainly been observed in subcortical structures (caudate nucleus and basal ganglia), connecting to the frontal cortical regions and forming a “fronto-striatal circuit” [17,64]. More specifically, an increased volume of the left caudate nucleus was associated with self-injurious behavior in boys with ASD, while compulsive and ritualistic behaviors showed significant positive correlations with bilateral caudate nuclei volumes [64]. Additionally, an increased functional connectivity between the left nucleus accumbens and a cluster in the right premotor cortex/middle frontal gyrus was related to more severe symptoms of repetitive behavior in children and adolescents with ASD [65]. Here, we report there is also an association between intra-individual variations in RRBs and changes in CT also in cortical regions that include the postcentral, parietal, frontal, and temporal regions in ASD. Our findings are thus in agreement with previous results in suggesting a crucial role of the fronto-striatal neurocircuitry in mediating RRB symptoms across development. We further extend these findings by reporting a correlation between the cortical aspect of this circuitry, indicating the importance of dysfunctional cortico-striatal connectivity due to atypical changes in cortical thickness in adolescents with ASD. 

Last, we aimed to link the macroscopic differences we observed at the neuroanatomical level to potential genomic mechanisms that have previously been linked to ASD. So far, few studies have examined the genetic and molecular mechanisms underpinning CT differences in ASD. A recent study by Romero-Garcia et al. (2019) demonstrated that differences in cortical thickness (CT) during childhood were robustly associated with genes involved in synaptic transmission pathways, which are known to be downregulated in the postmortem ASD cortex [42,66]. Similar results were also recently reported by our group when examining CT differences in ASD in a large and clinically heterogeneous sample [67]. The present results converge with these previous findings, as we observed an enrichment for genes and coexpressed modules that are known to be downregulated in ASD. ASD downregulated modules M16 and M10 are known to be involved in neuronal activity and synaptic function [42], which may contribute to deficits in synaptic-pruning and reduced cortical thinning [68]. Moreover, many ASD risk genes are likely to affect the maturation of excitatory and inhibitory neuronal pathways [44]. Our neuroimaging findings are also in line with genetic studies reporting a dysregulation of genes involved in the synaptic transmission of excitatory neurons across cortical layers (L2, L3, L4; [45]), and substantiate prior reports of an impaired excitatory–inhibitory balance underlying autism phenotypes [69]. We found no significant enrichment for genes previously linked to repetitive behaviors in ASD [46]. However, the RRB gene lists provided by Tao et al. (2016) are relatively small, and unspecific with regard to their functional involvement [46], and their functional role in brain development remains to be established. Overall, however, our finding of an enrichment for ASD-related gene sets adds to the biological plausibility of our neuroimaging findings, and links atypical CT development to specific etiological mechanisms in ASD.

Taken together, these three data modalities (CT change in ASD, RRB change in ASD, gene enrichment in ASD) have predominantly been studied in isolation. Our study is therefore among the first to use a longitudinal design to associate CT trajectories in adolescents with ASD to their development of RRBs, and to add specific genetic, and more precisely transcriptomic underpinning. 

The present study needs to be interpreted in the light of several limitations. First, our strict exclusion criteria and MRI quality assessment resulted in a comparatively small sample size. Larger studies of longitudinal samples are therefore needed to replicate our findings. Moreover, although RRBs are a common clinical feature of ASD, they are not unique to this condition [70]. Future studies linking developmental changes in repetitive behaviors to intra-individual variability in brain structure, using a transdiagnostic approach, are therefore needed to establish whether the observed brain–behavioral correlations are specific to ASD or generalized across mental health conditions. Moreover, here we focused on cortical regions. However, previous studies have also linked RRBs to subcortical regions, including the striatum [71,72]. Therefore, additional neuroimaging studies should extend the set of brain regions when characterizing the neuroanatomy underpinning RRBs. Last, the AHBA currently provides the most extensive source of anatomic and genomic information. However, it is based on adult donors. While we examined some adult participants (up to 18 years at T1 and up to 21 at T2), the AHBA does not fully cover our age range (11–21 years). We thus acknowledge the importance of repeating our analyses using gene expression data from young adults, once these become available.

## Figures and Tables

**Table 1:** Sample Characteristics.

	ASD (*n* = 33)	TD Controls (*n* = 37)	Group Comparison		
			*t*	*x* ^2^	*p*
Age [years]					
T1	14.48 ± 2.51	13.76 ± 2.36	1.25		0.22
T2	16.61 ± 2.47	15.89 ± 2.45	1.21		0.23
Interscan-Interval (ISI) [years]	2.07 ± 0.28	2.06 ± 0.20	0.17		0.87
Sex (male/female)	27/6	30/7		<0.001	1.00
Full-scale IQ (FSIQ)	101.38 ± 13.19	107.07 ± 11.52	−1.91		0.06
Handedness (right/left)	29/4	34/3		0.025	0.87
ADI-R *Social Interaction*	17.06 ± 4.96	-			
ADI-R *Communication*	13.06 ± 4.12	-			
ADI-R *Repetitive Behaviors*	4.76 ± 2.50	-			
ADOS *CSS*					
T1	5.79 ± 2.71	-			
T2 ^1^	5.95 ± 2.22	-			
RBS-R total					
T1	25.18 ± 18.80	2.11 ± 3.67	6.9		<0.001 ***
T2	22.48 ± 17.21	2.30 ± 4.07	6.58		<0.001 ***
∆(T2-T1)	−2.70 ± 16.00	+0.19 ± 3.01	−1.02		0.3148
RBS-R *persistent*					
T1	16.88 ± 11.26	1.59 ± 2.85	7.59		<0.001 ***
T2	14.97 ± 10.39	1.81 ± 03.23	6.98		<0.001 ***
∆(T2-T1)	1.91 ± 9.43	−0.22 ± 2.27	1.26		0.22
RBS-R *stereotyped*					
T1	3.06 ± 3.18	0.30 ± 0.62	4.91		<0.001 ***
T2	2.70 ± 3.66	0.08 ± 0.28	4.09		<0.001 ***
∆(T2-T1)	0.36 ± 3.63	0.22 ± 0.67	0.23		0.82
RBS-R *self-injurious*					
T1	0.88 ± 2.16	0.05 ± 0.23	2.18		<0.001 ***
T2	1.06 ± 1.84	0.03 ± 0.16	3.22		<0.001 ***
∆(T2-T1)	−0.18 ± 2.51	0.03 ± 0.29	−0.48		0.64
RBS-R *compulsive*					
T1	4.36 ± 5.17	0.16 ± 0.60	4.64		<0.001 ***
T2	3.76 ± 4.57	0.38 ± 1.14	4.14		<0.001 ***
∆(T2-T1)	0.61 ± 3.17	−0.22 ± 0.71	1.46		0.15
Total Brain Volume [l]					
T1	1.18 ± 1.00	1.24 ± 0.94	−2.60		<0.05 *
T2	1.17 ± 0.10	1.23 ± 0.10	−2.49		<0.05 *
∆(T2-T1)	−0.01 ± 0.02	−0.01 ± 0.02	−0.20		0.8445
Total Surface Area [m^2^]					
T1	0.18 ± 0.015	0.19 ± 0.02	−2.33		<0.05 *
T2	0.18 ± 0.16	0.19 ± 0.02	−2.28		<0.05 *
∆(T2-T1)	−0.02 ± 0.002	−0.002 ± 0.002	−0.16		0.877
Mean Cortical Thickness [mm]					
T1	2.75 ± 0.10	2.78 ± 0.08	−1.15		0.2555
T2	2.68 ± 0.08	2.70 ± 0.08	−0.81		0.4221
∆(T2-T1)	−0.07 ± 0.05	−0.08 ± 0.04	0.89		0.3759

Note: Data expressed as mean ± standard deviation; Abbreviations: ASD: Autism Spectrum Disorder, TD: Typical Developing, T1: timepoint 1, T2: timepoint 2, ADI-R: Autism Diagnostic Interview-Revised [29], RBS-R: Repetitive Behavior Scale-Revised [20,21], ADOS: Autism Observation Schedule, *CSS*: Calibrated Severity Score [33], mm: millimeter, l: liter, *m*^2^: square meter, *t*: t-value, *x^2^*: Pearson’s chi-squared test, *p:* *p* value, ***: *p* < 0.001, *: *p* < 0.05; ^1^ data based on *n* = 21 individuals.

**Table 2:** Clusters with significant between-group differences in the estimated developmental change in cortical thickness (CT_spc_) from T1 to T2.

Contrast	Cluster	Region Labels	Hemisphere	BA	Vertices	Talairach			*t*_max_	*p*_cluster_
						x	y	z		
ASD > Control										
	1	Superior frontal gyrus, medial orbital frontal cortex, rostral anterior cingulate cortex	R	4, 6, 8, 10, 11, 24, 32, 33	2358	10	50	11	5.07	9.59 × 10^−5^
	2	Lateral orbital frontal cortex, rostral middle frontal gyrus	R	9–11, 45–47	1716	25	44	−10	3.99	8.27 × 10^−4^
	3	Superior frontal gyrus	L	4, 6, 8, 10	1662	−16	37	41	3.13	9.83 × 10^-−4^
	4	Fusiform gyrus, parahippocampal gyrus, inferior temporal gyrus	L	20, 28, 34–37	1374	−23	−24	16	2.94	4.83 × 10^−3^
	5	Lateral orbital frontal cortex, rostral middle frontal gyrus, pars orbitalis	L	6, 8–11, 45–47	966	−22	43	−12	3.32	1.87 × 10^−2^
	6	Middle temporal gyrus	L	21	1318	−48	−13	−14	3.01	2.18 × 10^−2^
ASD < Control										
	7	Insula	L	13	580	−35	−4	−6	−1.67	3.84 × 10^−2^

Note: Hemisphere: L: Left, R: Right; BA: approximate Brodmann area(s); ASD: Autism Spectrum Disorder; Vertices: number of vertices within the cluster; *t*_max_: maximum *t*-statistic within the cluster; *p*-cluster: cluster-corrected *p* value.

## Data Availability

Data and code will be made available via request to the authors.

## Supplementary Materials

The following are available online at https://www.mdpi.com/article/10.3390/genes12122024/s1, Figure S1: Distribution of longitudinal changes in the RBS-R subdomain and total severity across groups, Figure S2: Correlation between symptom severity domains and group-differences in the symmetrized percent change in cortical thickness (CT_spc_), Figure S3: Interaction effect of longitudinal RBS-R changes-by-group on longitudinal changes in cortical thickness, Figure S4: Correlation between the symmetrized percent change in CT (CTspc) in ASD and the change in restricted and repetitive behavior (RRB), Figure S5: Significant between-group differences in cortical thickness estimates in scans that did not vs. did undergo the longitudinal stream in FreeSurfer, Figure S6: Genomic underpinnings of the main effect of RBS-R, Table S1: Overview of exclusion reasons, Table S2: Intra-individual differences in RBS-R total severity scores over time, Table S3: Main effect of a longitudinal change in RBS-R total severity on a longitudinal change in measures of cortical thickness. Supplementary Method 1: Sample description, including in- and exclusion criteria, Supplementary Method 2: Image processing and Quality Assessment, Supplementary Method 3: Model fitting using nested model comparison, Supplementary Method 4: Gene decoding analysis (GEDA), Supplementary Method 5: Enrichment analysis.
