# Supplementary material for: Longitudinal Changes in Cortical Thickness in Adolescents with Autism Spectrum Disorder and Their Association with Restricted and Repetitive Behaviors
Source: Genes (Basel). 2021 Dec 20;12(12):2024. doi: 10.3390/genes12122024 (PMC8701312; doi:10.3390/genes12122024)
Supplement: Supplementary file 1 [file genes-12-02024-s001.zip › Supplement_VB.pdf]

Supplementary information

Supplementary Methods **1**
1. Sample description, including in- and exclusion criteria 2
2. Image processing and Quality Assessment 3
3. Model fitting using nested model comparison 4
4. Gene decoding analysis (GEDA) 5
5. Enrichment analysis 6

Supplementary Figures **7**
Supplementary Figure S1 8
Supplementary Figure S2 9
Supplementary Figure S3 10
Supplementary Figure S4 11
Supplementary Figure S5 12
Supplementary Figure S6 13

Supplementary Tables **14**
Supplementary Table S1 15
Supplementary Table S2 16
Supplementary Table S3 17

Supplementary Methods

1. Sample description, including in- and exclusion criteria

We included children and adolescents aged between 11 and 18 years at timepoint one (T1) with a current clinical diagnosis of autism spectrum disorder (ASD) based on DSM-5 or ICD-10 criteria [1, 2]. All individuals with ASD met diagnostic cutoffs in the reciprocal social interaction (cutoff = 10), communication (cutoff = 8), and repetitive behaviors domain (cutoff = 3) of the Autism Diagnostic Interview-Revised (ADI-R) [3, 4]. The Autism Diagnostic Observation Schedule (ADOS-2) [5, 6] was conducted in all participants with ASD at their first assessment (T1), but only in 21 subjects at their second appointment (T2) and was not used as inclusion criteria. Depending on the subjects' age, different modules of the ADOS (i.e. module 3 and module 4) were applied between individuals and within individuals across timepoints.

To allow comparability of ADOS total severity and subdomain scores, ADOS Calibrated Severity Scores (CSS) were calculated [7]. To estimate overall intellectual ability, the Wechsler Abbreviated Scale of Intelligence (WASI-I) [8] was conducted at T1 as a short version of the Wechsler Intelligence Scales for Children (WISC-IV) [9] or Wechsler Adult Intelligence Scale (WAIS-IV) [10], providing indices of verbal, non-verbal, and full-scale IQ (FSIQ). We excluded participants with FSIQ < 70. As an exception, one female in the ASD group with FSIQ = 67 was included, to ensure groups were as gender-balanced as possible.

Further, this study contained the following exclusion criteria for all participants: contraindications to MRI, a history of major psychiatric or developmental disorder (e.g. psychosis), head injury, genetic disorders associated with ASD (e.g. fragile-X syndrome, tuberous sclerosis), as well as any medical condition affecting brain morphometry and function. In this study we included one participant with epilepsy. However, abnormalities in brain anatomy that may cause this disease or vice versa can be differentiated from those who can be related to behavioral patterns of ASD, due to the longitudinal study design. Furthermore, we excluded participants with a history of any drug abuse (including alcohol). Given the high number of individuals with ASD taking regular medication [11–13], participants on stable medication were, however, included.

2. Image processing and Quality Assessment

Image processing and cortical reconstruction were initially performed for N= 210 MRI scans (i.e. scans from n=105 subjects at timepoint 1 (T1) and timepoint 2 (T2), respectively) from an ongoing longitudinal study following the longitudinal stream in FreeSurfer (https://surfer.nmr.mgh.harvard.edu/fswiki/LongitudinalTwoStageModel). Here, models of the cortical surface are created for each T1-weighted image, i.e. one at T1 and one at T2 for each subject. These images are then used to create an unbiased within-subject template (i.e. base) that represents the average anatomy of each participant across time. The longitudinally processed cross-sectional images are subsequently derived by aligning the subjects' cross-sectional scans at each timepoint with the base. Stringent and standardized quality assessments were performed for all scans, i.e. five scans per subject (cross_T1, cross_T2, base, long_T1, long_T2) to ensure maximum data quality. Initially, all scans were visually examined by three independent raters, who could either (0) reject 'as is' (n= 12 or 2.29%), mostly due to severe (motion) artefacts and/or the existence of extra-brain tissue that precluded a successful FreeSurfer reconstruction, (1) accept a reconstruction 'as is' (n= 457 or 87.05%), or (2) prescribe manual editing (n= 56 or 10.67%) in case of smaller (i.e. 'local') reconstruction errors. We performed edits to the pial surface in case of interference of parts of the dura mater, skull or blood vessels with surface formation, which were erroneously identified as grey matter (gm). Segmentation errors of the white matter (wm) required edits to the white surface. Gm edits where performed in the longitudinally processed cross-sectional data (i.e. long_T1 and long_T2 scans), while wm edits were performed in the base template. After manual edits were performed, the images were re-preprocessed and visually re-assessed, to assure that editing improved the data quality. All 45 manually edited scans benefited from the process and were therefore included in the statistical analysis. However, other individuals were excluded from the statistical analysis due to the onset of a psychiatric disease after T1 in TD controls, or to achieve a gender-balanced design across groups (see Supplementary Table S1). We excluded 12 female controls to receive an approximate gender ratio of ~ 4:1 in both samples. A ratio of 4:1 has been chosen to reflect the gender ratio widely cited in the literature, stating that ASD is diagnosed three to four times for often in males than in females [14–16] To obtain a similar male-to-female ratio in our study as in the general population, we have therefore specifically

recruited ASD individuals at a prevalence of 4:1. Since the focus of the study was on repetitive behaviors, we further excluded participants with missing RBS-R data. The final data set thus contained usable MRI data of n= 70 participants.

3. Model fitting using nested model comparison

The employed general linear model (GLM) was selected after we initially calculated vertex-wise nested model comparisons (i.e. F-tests) to examine the goodness-of-fit of distinct model versions in the total sample, i.e. individuals with ASD and TD controls. The identification of the most parsimonious model was guaranteed by the use of a step-up model selection procedure. In this stepwise approach, a reduced model is compared to a more complex model with results indicating whether the addition of a new model term significantly increased the goodness-of-fit at each vertex. While the inclusion of the quadratic age term yielded an improved goodness-of-fit relative to the basic (i.e. reduced) model, all other examined variables, namely Age_{T1}-by-group and Age_{T1}^2-by-group, did not increase the goodness-of-fit.

To address significant variation in brain sizes between males and females, we corrected for sex in all analysis by including a "sex" term as covariate in our general linear model. Furthermore, the longitudinal pipeline of FreeSurfer computes *intra*-individual changes of brain structure rather than *inter*-individual differences. Values of spc (change in CT) are therefore by default corrected for differences in total brain size.

4. Gene decoding analysis

In order to decode the unthresholded *t*-maps of the main effect of group (Fig. 1a) and the main effect of change in RBS-R total severity from T1 to T2 (ΔRBS-$R_{(T2-T1)}$) in individuals with ASD on measures of spc (Figure 3b), derived from Matlab, we initially uploaded them to the NeuroVault server (https://neurovault.org). The gene decoding analysis (GEDA) was subsequently performed in R using code embedded in NeuroVault and Neurosynth (https://neurosynth.org). All 20,787 protein coding genes from the Allen Human Brain Atlas (AHBA; [17]) were tested for spatial gene expression that resembles our neuroimaging difference maps [18]. More specifically, the analysis constructs a linear model for each of the six donor brains from the AHBA, where the slopes encode how similar each gene's spatial expression pattern is to the input imaging map. In line with the input maps, these analyses are restricted to cortical tissue. The slopes are then subjected to a one-sample *t*-test to identify genes whose spatial expression patterns consistently, i.e. across the donor brains, show high resemblance to the imaging maps.

5. Enrichment analysis

To elucidate the biological function of the genes derived from the GEDA and their relevance in ASD, a gene enrichment analysis was computed using the GeneOverlap package in R (10.18129/B9.bioc.GeneOverlap). Here, we tested our GEDA gene-list, derived from the *t*-map displaying between-group differences (thresholded at $p < 0.05$), for enrichment with various gene-sets associated with ASD at genetic and transcriptomic level. For enrichment testing for genetic associations with ASD, we used the 102 rare and de novo protein truncating variants

identified in the largest exome sequencing study of ASD to date [19]. We also included an ASD-related gene list compiled by SFARI (SFARI.ASD.genes; categories S, 1, 2, & 3 downloaded in November 2020 from https://gene.sfari.org/). A list of differentially expressed genes (DEGs) (up-/downregulated) in post mortem tissue was further utilized to test for gene enrichment at the transcriptomic level [20], as well as for genes that are differentially expressed in specific cell types in ASD (astrocytes, excitatory and inhibitory neurons; [21]). Moreover, we included genes from differentially expressed co-expression modules in ASD that map onto specific biological processes, i.e. synaptic transmission [22].

For enrichment analysis of the gene list we derived by the GEDA of the *t*-map with the main effect of RBSR change in ASD (thresholded at $p < 0.05$) we employed a gene set that has been linked to Repetitive Sensory Motor behavior (RSM) and Insistence on Sameness (IS) in a genome-wide association study (GWAS; [23]). The study identified a significant association between RSM and IS with various Single Nucleotid Polymorphisms (SNPs), which are part of gene locus 8p21.2-8p21.1.

Supplementary Figures

Supplementary Figure S1. Distribution of longitudinal changes in RBS-R subdomain and total severity across groups

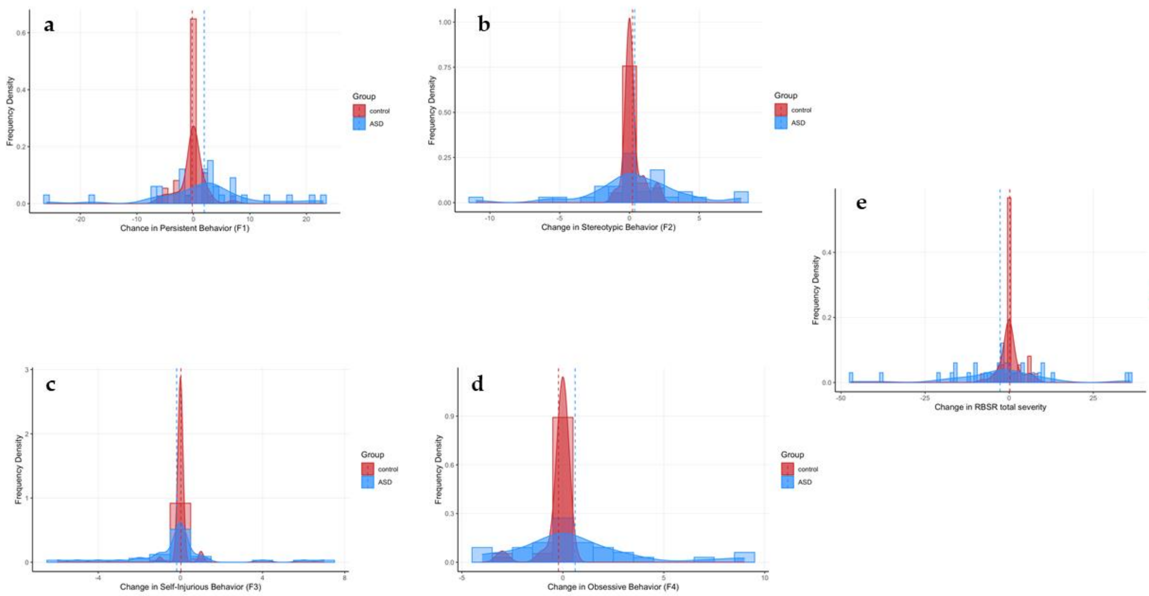

Histograms displaying the distribution of longitudinal changes in subscale (i.e. persistent (A), stereotyped (B), obsessive (C), and self-injurious (D) behavior) and total severity raw scores in the German version of the Repetitive Behavior Scale-Revised (RBS-R) in individuals with autism spectrum disorder (ASD; blue) and typically developing controls (red).

Supplementary Figure S2. Correlation between symptom severity domains and group-differences in symmetrized percent change (spc) of CT

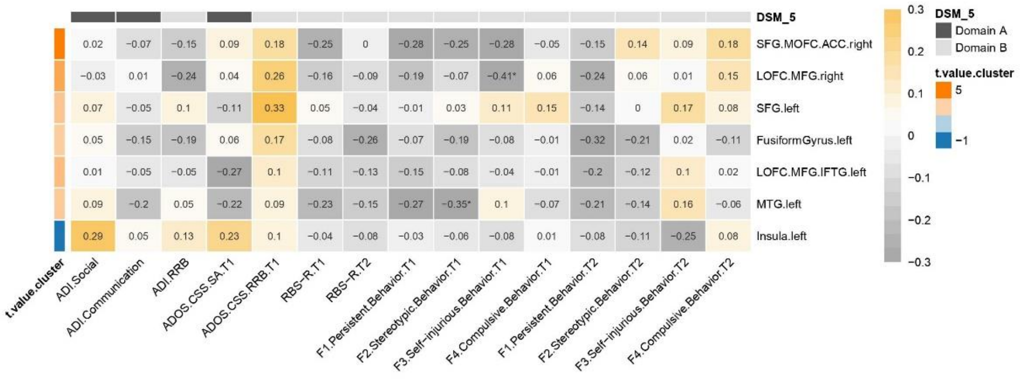

Brain-behaviour correlations between clusters with significant differences in symmetrized percent change of cortical thickness (CT_{spc}) and measures of symptom severity subdivided into DSM-5 Domain A and B symptoms within the ASD group. We observed a significant negative correlation (*) between CTspc in the right lateral orbital frontal cortex and right rostral middle frontal gyrus and a change in self-injurious behavior as measured by the RBS-R. We also observed a significant negative correlation between CTspc and a change in stereotypic behavior in the left middle temporal gyrus. Right: right hemisphere, left: left hemisphere, SFG: superior frontal gyrus, MOFC: medial orbital frontal cortex, ACC: anterior cingulate cortex, LOFC: lateral orbital frontal cortex, MFG: middle frontal gyrus, IFTG: inferior temporal gyrus, MTG: middle temporal gyrus, tAIs: subject-level total neuroanatomical abnormality index, ADI: Autism Diagnostic Interview-Revised, RRB: repetitive/restricted behaviour, ADOS.SA/ADOS.RRB: Autism Diagnostic Observation Schedule Calibrated Severity Score for Social Affect (SA) and restricted and repetitive behaviours (RRB), SRS-2: Social Responsiveness Scale-2, RBS-R: Repetitive Behaviors Scale – Revised.

Supplementary Figure S3. Interaction effect of longitudinal RBS-R changes-by-group on longitudinal changes in cortical thickness

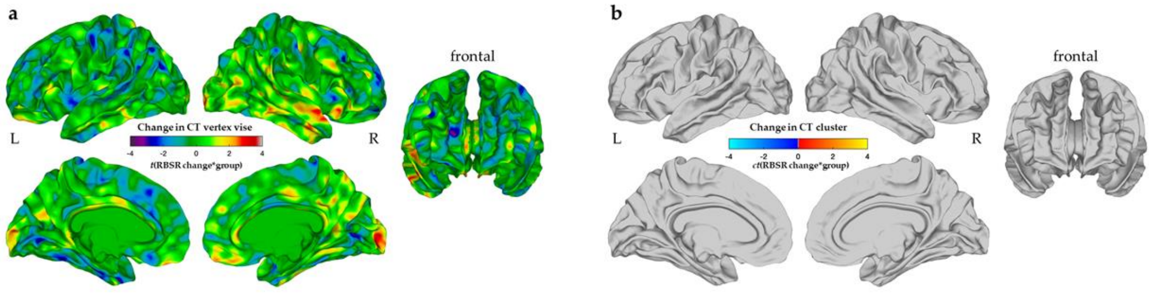

Interaction effect of longitudinal changes in total severity on the German version of the Repetitive Behavior Scale-Revised (RBS-R)-by-group (i.e. individuals with autism spectrum disorder vs. typically developing controls) on longitudinal changes in cortical thickness, quantified by the symmetrized percent change (spc; https://surfer.nmr.mgh.harvard.edu/fswiki/LongitudinalTwoStageModel). Displayed are the un-thresholded *t*-maps (a) and the random field theory (RFT)-based cluster corrected ($p<0.05$, 2-tailed) difference maps following multiple comparisons (b). *Abbreviations:* L: left hemisphere, R: right hemisphere.

Supplementary Figure S4. Correlation between symmetrized percent change in CT (CT_{spc}) in ASD and change in restricted and repetitive behavior (RRB).

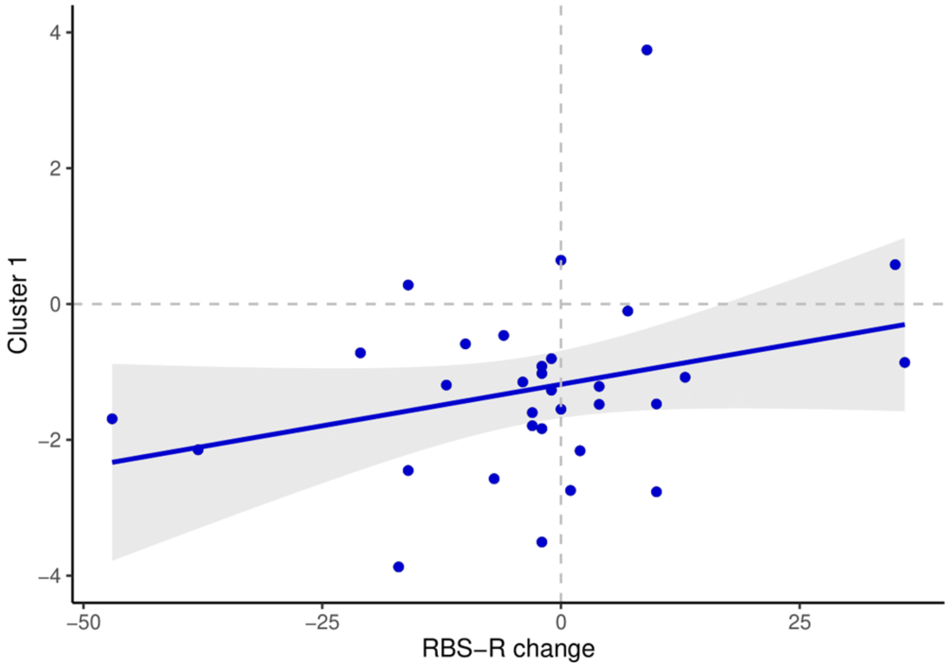

Correlation plot of CT_{spc} in significant Cluster 1 (y-axis; right superior temporal gyrus, middle temporal gyrus, inferior parietal cortex, banks superior temporal sulcus, lateral occipital cortex and transverse temporal cortex) and change in RRB (x-axis) measured by the Restricted and Repetitive Behavior Scale Revised (RBSR) total score. Negative CT_{spc} values indicating cortical thinning are most strongly correlated with decreasing RBSR total scores reflecting an improvement in RRB symptom severity.

Supplementary Figure S5. Significant between-group differences in cortical thickness estimates in scans that did not vs. did undergo the longitudinal stream in FreeSurfer

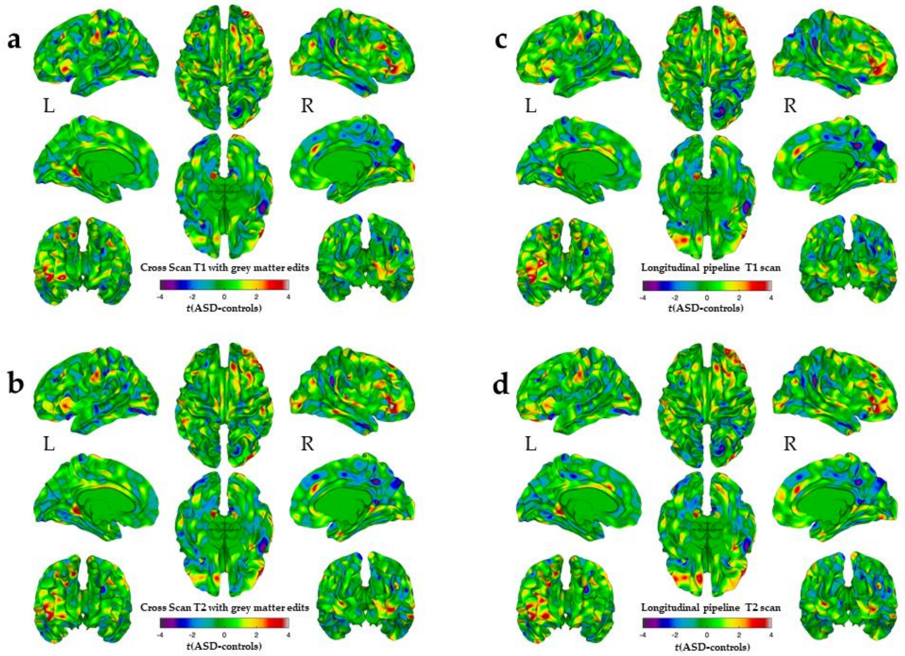

Vertex-wise between-group differences in cortical thickness (CT) estimates in individuals with autism spectrum disorder (ASD) relative to typically developing (TD) controls in cross-sectionally processed MRI data at timepoint 1 (T1) (a) and timepoint 2 (T2) (b) compared to cross-sectional scans at T1 (c) and T2 (d) that were processed using longitudinal stream in FreeSurfer [24]. Displayed are the un-thresholded *t*-maps (a-d), where increased CT in individuals with ASD compared to TD controls is marked in yellow to red and reduced CT is marked in blue to cyan. *Abbreviations:* L: left hemisphere, R: right hemisphere.

Supplementary Figure S6. Genomic underpinnings of the main effect of RBSR

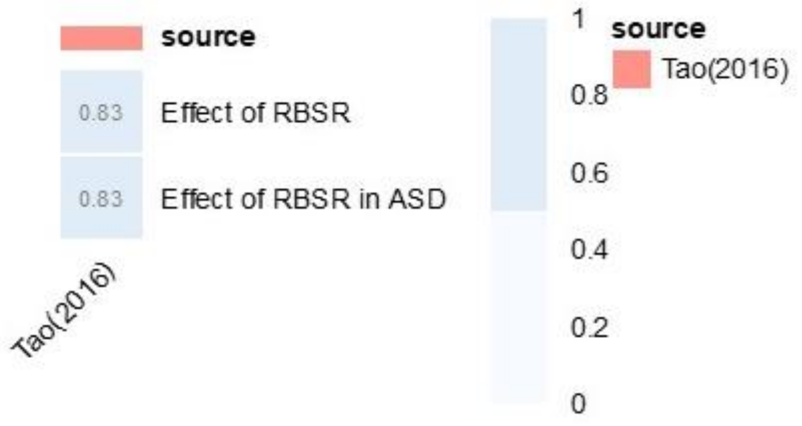

Genomic underpinnings of the effect of the Restricted and Repetitive Behavior Scale-Revised (RBSR) total score on symmetrized percent change in cortical thickness (CT_{spc}) based on the *t*-map of (1) the main effect of RBSR in the total sample (n=70) and (2) in the ASD sample (n=33). For the enrichment analysis a gene list with gene expression patterns known to be associated with restricted and repetitive behavior of Tao et al. (2016) was used [23]. No Significant Odds-ratios (OR) at an FDR-rate *p*-value <0.05 resulted for genes expressed in the *t*-maps.

Supplementary Tables

Supplementary Table S1. Overview of exclusion reasons

	ASD	controls
**N**	10	25
Psychiatric diagnosis[1]	0	4
Premature birth	0	1
Missing RBS-R data	6	8
Abnormalities in brain anatomy[2]	2	0
Bad qualitiy of MRI scans[3]	2	0
Matching samples[4]	0	12

Note: [1]Comorbid diagnosis were allowed in individuals with autism spectrum disorder (ASD), e.g. Attention-deficit/hyperactivity disorder (ADHD), functional eneuresis, mild depressive episode, mixed obsessional thoughts and acts; [2]cysts; [3]motion artefacts; [4]exluding 12 female controls, to achieve gender-balanced sample; *Abbreviations:* RBS-R: Repetitive Behavior Scale-Revised; MRI: magnetic resonance imaging.

Supplementary Table S2. Intra-individual differences in RBS-R total severity scores over time

	Increase (RBSR total score)				Decrease (RBSR total score)				Stable in RBSR total
	Mean	Standard deviation	%	MaxI	Mean	Standard deviation	%	MaxD	%
ASD	+ 11	± 12	33.3	+ 35	- 11	± 12	60.1	- 47	6
controls	+ 4	± 2	21.2	+ 8	- 4	± 2	21.6	- 8	57

Note: Based on the total sample (n=70); [%] = percentage of individuals in the ASD or control group with an increase/decrease or stable values of RBSR-total score; [MaxI] = maximum increase in RBSR total score; [MaxD] = maximum decrease in RBSR total score; RBSR: Restricted and repetitive-Behavior-Scale-Revised.

Supplementary Table S3. Main effect of longitudinal change in RBS-R total severity on longitudinal change in measures of cortical thickness

Contrast	Cluster	Region Labels	Hemisphere	BA	Vertices	Talairach x	y	z	t_{max}	$p_{cluster}$
ΔRBS-R$_{(T2-T1)}$										
	1	precuneus cortex, isthmus-cingulate cortex	R	7, 26, 29-31	2335	9	-49	23	3.36	6.76×10^{-4}
	2	supramarginal gyrus	R	40	2192	47	-17	19	3.79	6.78×10^{-4}

	3	superior frontal gyrus, caudal anterior-cingulate cortex	R	4, 6, 8, 10, 24, 33	1933	7	33	15	4.48	1.01×10^{-3}
	4	precuneus cortex, isthmus-cingulate cortex	R	7, 26, 29-31	1465	21	-53	10	3.53	3.46×10^{-3}
	5	superior temporal gyrus, middle temporal gyrus	R	20-22, 42	2223	48	-17	-7	3.41	4.29×10^{-3}
	6	lingual gyrus, fusiform gyrus	R	17-19, 37	1039	21	-68	0	2.84	3.70×10^{-2}

Note: Based on the total sample (n= 70), i.e. individuals with autism spectrum disorder (ASD) and typically developing (TD) controls. Hemisphere: *L*: Left, *R*: Right; *BA*: approximate Brodmann area(s); Vertices: number of vertices within the cluster; t_{max}: maximum *t*-statistic within the cluster; *p*-cluster: cluster-corrected *p*-value.

**Contrast**	**Cluster**	**Region Labels**	**Hemisphere**	**BA**	**Vertices**	**Talairach**			t_{max}	$p_{cluster}$
						**x**	**y**	**z**		
ΔRBS-R$_{(T2-T1)}$										
	1	superior temporal gyrus, middle temporal gyrus, inferior parietal cortex, banks superior temporal sulcus, lateral occipital cortex, transverse temporal cortex	R	5, 7, 17-22, 41, 42	5948	65	-26	6	4.00	4.28×10^{-5}
	2	lateral orbital frontal cortex, precuneus cortex, isthmus-cingulate cortexortex, rostral middle frontal gyrus	R	7, 11, 26, 29-31, 46	4283	6	-39	30	4.25	6.52×10^{-5}
	3	supramarginal gyrus, postcentral gyrus, insula	R	1-3, 13, 40	3391	43	-20	19	3.61	6.40×10^{-4}

Note: Based on individuals with autism spectrum disorder (ASD) only (n= 33). Hemisphere: *L*: Left, *R*: Right; *BA*: approximate Brodmann area(s); Vertices: number of vertices within the cluster; t_{max}: maximum *t*-statistic within the cluster; *p*-cluster: cluster-corrected *p*-value.

References

[1] American Psychiatric Association, *Diagnostic and statistical manual of mental disorders (5th Ed): DSM-5*, 5th ed. Washington DC: American Psychiatric Association, 2013.

[2] H. Dilling, W. Mombour, M. H. Schmidt, E. Schulte-Markwort, and H. Remschmidt, Eds., *Internationale Klassifikation psychischer Störungen: ICD-10 Kapitel V (F) klinisch-diagnostische Leitlinien*, 10th ed. Bern: Hogrefe Verlag, 2015.

[3] C. Lord, M. Rutter, and A. Le Couteur, "Autism Diagnostic Interview-Revised: a revised version of a diagnostic interview for caregivers of individuals with possible pervasive developmental disorders," *Journal of autism and developmental disorders*, vol. 24, no. 5, pp. 659–685, 1994, doi: 10.1007/BF02172145.

[4] S. Bölte, M. Rutter, A. Le Couteur, and C. Lord, *ADI-R - diagnostisches Interview für Autismus - revidiert: Manual*. Bern: H. Huber; zu beziehen bei Testzentrale der Schweizer Psychologen etc, 2006.

[5] L. Poustka, D. Rühl, S. Feineis-Matthews, S. Bölte, F. Poustka, and M. Hartung, *ADOS-2. Diagnostische Beobachtungsskala für Autistische Störungen -2*.

[6] C. Lord, M. Rutter, R. J. Luyster, and K. Gotham, *Autism Diagnostic Observation Schedule, 2nd Edition*.

[7] K. Gotham, A. Pickles, and C. Lord, "Standardizing ADOS scores for a measure of severity in autism spectrum disorders," *Journal of autism and developmental disorders*, vol. 39, no. 5, pp. 693–705, 2009, doi: 10.1007/s10803-008-0674-3.

[8] D. Wechsler, "Wechsler abbreviated scale of intelligence (WASI) manual.: San Antonio: TX: Psychological Corporation;," vol. 1999.

[9] F. Petermann and U. Petermann, Eds., *Wechsler intelligence scale for children - fourth edition: Manual 1: Grundlagen, Testauswertung und Interpretation : Übersetzung und Adaptation der WISC-IV® von David Wechsler*, 2nd ed. Frankfurt/M.: Pearson, 2014.

[10] D. Wechsler, *Wechsler Adult Intelligence Scale–Fourth Edition (WAIS-IV)*. San Antonio, TX: NCS Pearson., 2008.

[11] C. M. Freitag *et al.*, "Group-based cognitive behavioural psychotherapy for children and adolescents with ASD: the randomized, multicentre, controlled SOSTA-net trial," *Journal of child psychology and psychiatry, and allied disciplines*, vol. 57, no. 5, pp. 596–605, 2016, doi: 10.1111/jcpp.12509.

[12] A. Y. S. Wong *et al.*, "The variation of psychopharmacological prescription rates for people with autism spectrum disorder (ASD) in 30 countries," *Autism research : official journal of the International Society for Autism Research*, vol. 7, no. 5, pp. 543–554, 2014, doi: 10.1002/aur.1391.

[13] T. W. Frazier, P. T. Shattuck, S. C. Narendorf, B. P. Cooper, M. Wagner, and E. L. Spitznagel, "Prevalence and correlates of psychotropic medication use in adolescents with an autism spectrum disorder with and without caregiver-reported attention-deficit/hyperactivity disorder," *Journal of child and adolescent psychopharmacology*, vol. 21, no. 6, pp. 571–579, 2011, doi: 10.1089/cap.2011.0057.

[14] R. Loomes, L. Hull, and W. P. L. Mandy, "What Is the Male-to-Female Ratio in Autism Spectrum Disorder? A Systematic Review and Meta-Analysis," *Journal of the American Academy of Child and Adolescent Psychiatry*, vol. 56, no. 6, pp. 466–474, 2017, doi: 10.1016/j.jaac.2017.03.013.

[15] D. M. Werling and D. H. Geschwind, "Sex differences in autism spectrum disorders," *Current opinion in neurology*, vol. 26, no. 2, pp. 146–153, 2013, doi: 10.1097/WCO.0b013e32835ee548.

[16] E. Fombonne, "Epidemiology of pervasive developmental disorders," *Pediatric research*, vol. 65, no. 6, pp. 591–598, 2009, doi: 10.1203/PDR.0b013e31819e7203.

[17] M. J. Hawrylycz *et al.*, "An anatomically comprehensive atlas of the adult human brain transcriptome," *Nature*, vol. 489, no. 7416, pp. 391–399, 2012, doi: 10.1038/nature11405.

[18] K. J. Gorgolewski *et al.*, "Tight fitting genes: finding relations between statistical maps and gene expression patterns," *F1000Research*, vol. 5, 2014, doi: 10.7490/f1000research.1097120.1.

[19] F. K. Satterstrom *et al.*, "Large-Scale Exome Sequencing Study Implicates Both Developmental and Functional Changes in the Neurobiology of Autism," *Cell*, vol. 180, no. 3, 568-584.e23, 2020, doi: 10.1016/j.cell.2019.12.036.

[20] M. J. Gandal *et al.*, "Shared molecular neuropathology across major psychiatric disorders parallels polygenic overlap," *Science (New York, N.Y.)*, vol. 359, no. 6376, pp. 693–697, 2018, doi: 10.1126/science.aad6469.

[21] D. Velmeshev *et al.*, "Single-cell genomics identifies cell type-specific molecular changes in autism," *Science (New York, N.Y.)*, vol. 364, no. 6441, pp. 685–689, 2019, doi: 10.1126/science.aav8130.

[22] N. N. Parikshak *et al.*, "Genome-wide changes in lncRNA, splicing, and regional gene expression patterns in autism," *Nature*, vol. 540, no. 7633, pp. 423–427, 2016, doi: 10.1038/nature20612.

[23] Y. Tao, H. Gao, B. Ackerman, W. Guo, D. Saffen, and Y. Y. Shugart, "Evidence for contribution of common genetic variants within chromosome 8p21.2-8p21.1 to restricted and repetitive behaviors in autism spectrum disorders," *BMC genomics*, vol. 17, p. 163, 2016, doi: 10.1186/s12864-016-2475-y.

[24] M. Reuter, N. J. Schmansky, H. D. Rosas, and B. Fischl, "Within-subject template estimation for unbiased longitudinal image analysis," *NeuroImage*, vol. 61, no. 4, pp. 1402–1418, 2012, doi: 10.1016/j.neuroimage.2012.02.084.
